# 

**Published:** 1858-01

**Authors:** 


### CAUSE OF THE

### COAGULATION OF THE BLOOD:

WITH A PRACTICAL APPENDIX.

BY

BENJAMIN W. RICHARDSON, M.D., L.R.C.P.,

PHYSICIAN TO THE ROYAL INFIRMARY FOR DISEASES OF THE CHEST.

---

**Contents:**

CHAPTER I. Historical Memoranda on the supposed Causes of Coagulation of the Blood.

CHAPTER II.—Cause of the Phenomenon of Coagulation: present position of the Question.

CHAPTER III.—The Author's Personal Observations and Deductions on the Condition of the Blood in the Body under certain Physiological and Pathological States.

CHAPTER IV.—Experimental Inquiry into the Physical Agencies influencing the Coagulation of the Blood.

CHAPTER V.—Experimental Inquiry into the Chemical Agencies influencing the Coagulation of the Blood.

CHAPTER VI.—Experimental Inquiry into the Chemical Agencies influencing the Coagulation of the Blood (continued).

CHAPTER VII.—Phenomenon of the Buffy Coat.

CHAPTER VIII.—Summary of Conclusions and Propositions.

APPENDIX.

1.—Superalkaline Conditions of the Blood in Relation to Disease.

2.—Ammonia as an Exhalation from the Body.

3.—Experiments, shewing the Effect of Lactic Acid on Animal Bodies (with Coloured Plates, shewing the stages of Endocarditis).

4.—Deposition of Fibrin in the Heart and Blood-vessels during Life.

5.—The Alkalies and Acids as Remedial Agents.

6.—Application of Ammonia to the Experiment of Transfusion of Blood.

7.—On some Conditions of the Blood after Death, in relation to Medico-Legal Inquiries.

8.—List of Authors.

*The Essay is Illustrated with numerous Wood Engravings, and with Coloured Plates by Tuffen West.*

London: JOHN CHURCHILL, New Burlington Street.

### To New Subscribers to the Sanitary Review.

The Publisher begs to intimate, that new Subscribers wishing to have the series of the Journal complete from the first number, can obtain sets of Vols. I, II, and III, at Subscribers' prices, 10s. each, post free, by application to him, enclosing Post-office Order, payable at the Charing Cross Branch to Thomas Richards.

The Sanitary Review and Journal of Public Health is sold by all Booksellers, and is equally suited for the general and professional reader, for the scientific society, and for the private library.

London: T. Richards, 37, Great Queen Street.

**ORIGINAL COMMUNICATIONS:**

*On* 1*st January*, 1858, *will be published No.* 20, *being the fourth number of Vol. V. of the*

### GLASGOW MEDICAL JOURNAL;

EDITED BY

DRS. GEORGE BUCHANAN & JOHN B. COWAN.

**Contents:**

ORIGINAL COMMUNICATIONS.

I. The Philosophy of Apparitions.
II. On Shortening the duration of Labour. By James Gray, M.D.
III. On Aneurism of the Popliteal Artery. By M. S. Buchanan, M.D.
IV. On Pityriasis Rubra. By James McGhie, M.D.
V. The Medical Pilgrim's Progress; an Address to Students. By Benjamin W. Richardson, M.D., London.
VI. Compound Fracture of the Skull. By John Couper, Esq.

REVIEWS.

I. Aitkin's Practice of Physic.
II. Berkeley, Cryptogamic Botany.
III. Smith on Stricture of the Urethra.
IV. Turner's Atlas of Anatomy.
V. Parliamentary Report on Vaccination.

Quarterly Report of Cases treated in the Glasgow Royal Infirmary, &c.,
With a copious Index to the Volume.

Glasgow: WILLIAM MACKENZIE, 45 & 47, Vincent Street;
London: 72, Paternoster Row.

